# Direct Observation of Filling Process and Porosity Prediction in High Pressure Die Casting

**DOI:** 10.3390/ma12071099

**Published:** 2019-04-02

**Authors:** Hanxue Cao, Chao Shen, Chengcheng Wang, Hui Xu, Juanjuan Zhu

**Affiliations:** 1 Materials Forming and Control Department, College of Materials Science and Engineering, Chongqing University, Chongqing 400044, China; 20160902004@cqu.edu.cn (C.S.); 13883258439@163.com (C.W.); 2 National Engineering Research Center for Magnesium Alloys, Chongqing University, Chongqing 400044, China; 3 Chongqing Changan Automobile Co., Ltd., Chongqing 400023, China; xuhui1@changan.com.cn (H.X.); zhu10041004@163.com (J.Z.)

**Keywords:** direct observation, filling process, porosity prediction, high pressure die casting

## Abstract

Although numerical simulation accuracy makes progress rapidly, it is in an insufficient phase because of complicated phenomena of the filling process and difficulty of experimental verification in high pressure die casting (HPDC), especially in thin-wall complex die-castings. Therefore, in this paper, a flow visualization experiment is conducted, and the porosity at different locations is predicted under three different fast shot velocities. The differences in flow pattern between the actual filling process and the numerical simulation are compared. It shows that the flow visualization experiment can directly observe the actual and real-time filling process and could be an effective experimental verification method for the accuracy of the flow simulation model in HPDC. Moreover, significant differences start to appear in the flow pattern between the actual experiment and the Anycasting solution after the fragment or atomization formation. Finally, the fast shot velocity would determine the position at which the back flow meets the incoming flow. The junction of two streams of fluid would create more porosity than the other location. There is a transition in flow patterns due to drag crisis under high fast shot velocity around two staggered cylinders, which resulted in the porosity relationship also changing from R1 < R3 < R2 (0.88 m/s) to R1 < R2 < R3 (1.59 and 2.34 m/s).

## 1. Introduction

The high pressure die casting (HPDC) process has the characteristics of high speed filling and high pressure solidification, which promotes it to become one of the main manufacturing processes of complicated thin-wall components [1]. Mold has enormous effect on the quality of aluminum alloy die-castings, such as surface finish, productivity and microstructure refinement. The melt flow is controlled by the mold design during the filling process, and the parameters associated with the mold design need to be carefully considered: sprue, gates, mold positioning, mold lubrication, thickness to be filled and cooling system [2]. In addition, unreasonable gate system design will reduce the mold life due to aluminum soldering mechanisms. The treatments applied to the mold surface are extremely important to prolonging the lifespan of mold, such as TiAlN coatings [3]. A mold surface with excellent performance can facilitate the part extraction in the ejection phase and acquire the integrated parts with smooth surfaces. It is well known that porosity is the main defect in die-castings, and porosity can seriously damage mechanical properties of die-castings. Air entrapment in the liquid metal during the filling process is the major source of porosity [4]. Therefore, to obtain high-performance castings, it is extremely important to observe the die casting filling process and predict the air entrapment. Thus, the filling process can be improved, and the defects rate can be reduced by changing the gate system and venting system at the mold level according to melt flow observed in mold [5].

At present, computational fluid mechanics and experimental fluid mechanics are two main methods to study the filling process and predict gas entrapment defects. Computational fluid mechanics could predict the filling process through calculation and provide a powerful and cost effective tool to control the filling process of die casting, and many new models and methods are presented by many scholars in the HPDC field. Cao et al. [6] used the gas-liquid multiphase flow model to predict the air entrainment phenomenon during the die casting filling process of zinc alloy, and the volume of fluid (VOF) was used to track free surface. A water-filling experiment in an S-shaped channel was simulated, and the simulated results were basically consistent with the experimental results. Bi et al. [7] used the coupling of the level-set method and the VOF method to capture the free interface and obtain its normal vector, and used the continuous surface force (CSF) model to consider the surface tension, which was verified by experiment and could improve the simulation accuracy on interface geometries, liquid flows and gas entrapments.

In addition to the above grid method, the grid-free method has received more attention in recent years, especially smooth particle hydrodynamics (SPH). By solving the governing equation described by the Lagrangian method, the SPH method can better deal with fluid flow with significant free surface fragmentation and splashing during the die casting filling. Cleary et al. [8] used the SPH model to simulate the filling process of laptop chassis, and found that the results were highly consistent with the experiments results, indicating that the SPH model is very suitable for simulating complex thin-wall die casting. Nevertheless, liquid metal is injected into the die cavity at a high velocity (around 30~100 m/s) in HPDC. The resulting flow is transient and complex. Although the simulation accuracy makes progress rapidly, it is in an insufficient phase because of complicated phenomena of the filling process and difficulty of experimental verification in HPDC [9,10], especially in thin-wall complex die-castings. Therefore, it is hoped that the actual filling process could be observed directly so as to provide the reference to improve the accuracy of numerical simulation.

The simulation result adopted a new model and a new method should be validated experimentally. At the moment, the water analogue experiment is the most common method of experimental fluid mechanics to observe the filling process and to validate the numerical predictions [6,7,8,11,12]. However, although these experiments are designed according to dynamic similarity [13,14], water is not the actual melt. The physicochemical properties and surface tension of water is much different from the actual melt. Especially, surface tension plays an extremely important role in various surface and interface phenomena [7,15]. Moreover, the water analogue experiment is generally conducted at room temperature and does not take into account the temperature change, so it cannot really accurately capture the actual filling process. In addition, Ohnaka et al. [10] presented that there was another approach to directly observe the filling process during HPDC by X-ray diffraction. Although the filling process could be observed intuitively, an especially designed mold (graphite mold rather than thick steel mold) should be adopted due to the characteristics of the X-ray absorption image, and the equipment required is expensive.

In this paper, a new method is introduced to directly observe the actual die casting filling process with high speed, high temperature and high pressure. Three flow visualization experiments were conducted. The actual filling process is captured directly by high speed camera. Comparing the actual filling process observed and the numerical simulation solution, the differences of flow pattern between the two are obtained. Meanwhile, the porosity of die-castings is predicted at different locations according to the actual filling process. 

## 2. Materials and Methods

A horizontal cold chamber die casting machine (L.K. Technology Holdings Limited, Hong Kong, China) was used in this experiment. The casting material was 99.7% pure aluminum. Three flow visualization experiments were conducted. The first stage speed was 0.13 m/s, the second stage speed was 0.27 m/s and the fast shot set point was 270 mm. The fast shot speed was 0.88, 1.59 and 2.34 m/s, respectively. At the beginning of each test, the liquid metal was taken from the furnace at the temperature of 750 ± 10 °C, and the liquid metal was manually poured into the shot sleeve with an inner diameter of 70 mm and a length of 380 mm. The pouring time was about 4 sec. At that time, the liquid metal (melt volume: ~530,000 mm^3^) accounted for about 36% of the whole shot sleeve. The die (material: P20 steel) was heated to 200 °C before pouring the molten metal. Then, the plunger tip was rapidly accelerated to reach the desired injection velocity. The nominal pressure of the HPDC machine was 13.5 MPa. There was no pressure intensification stage in these experiments. 

**Figure 1:**
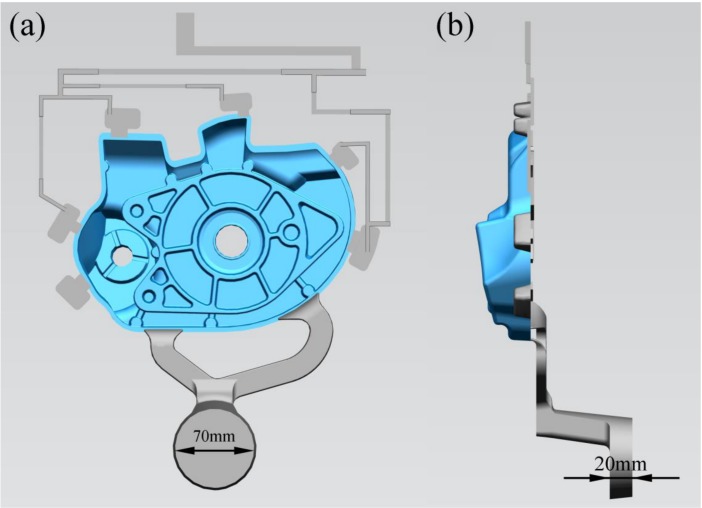
Geometry of die castings for the flow visualization experiment.

**Figure 2:**
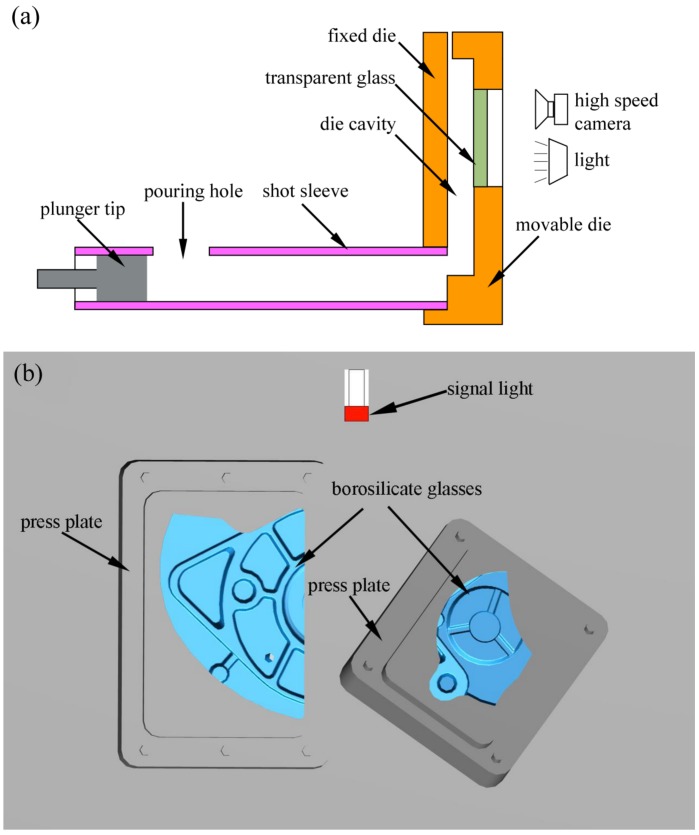
The flow visualization setup. (**a**) A schematic showing the flow visualization experiment setup; (**b**) the two shooting windows of high speed camera (the dimension of the bigger is 150 × 101 mm and the dimension of the smaller is 104 × 79 mm) on the movable die.

Figure 1 shows the geometry of die castings for the flow visualization experiment. Figure 2 shows a schematic of the flow visualization experimental setup used to record the flow patterns. The transparent borosilicate glasses were placed on the movable die to visualize the real time flow patterns during filling the die cavity. Table 1 shows the properties of borosilicate glass used. The position and size of the two transparent windows (the dimension of the bigger is 150 × 101 mm and the dimension of the smaller is 104 × 79 mm) is shown in Figure 2b, and the windows were parallel to the castings. The bottom of the left window is located in the inner gate, and the left window is a vertical plane, so the flow of aluminum liquid into the mold cavity can be observed conveniently. There are two staggered cylinders in the right window, and the flow of liquid aluminum around them could be studied under die casting conditions. Flow patterns of the aluminum melt were captured using a high speed camera with a sampling rate of 1000 frames per second and a shutter speed of 1/1000 seconds. To determine the start time of plunger tip moving in the video, a signal light was placed in the shooting area as shown in Figure 2b. As long as the plunger tip started to move, the signal light went on.

A popular commercially available software package (Anycasting software) (AnyCasting Co.Ltd., Seoul, Korea) for casting simulations and analysis was adopted. The CAD date of mold was imported into the software. The filling process simulation of the same process was conducted. The boundary conditions and mesh properties are listed in Table 2. The finite difference meshes were adopted in the Anycasting simulation. The SOLA-VOF (Solution Algorithm-Volume of Fluid) approach was used to solve the coupling between the momentum and mass conservation equations and to trace the free surface in Anycasting software. The thermophysical properties of melt used in the simulation are listed in Table 3. The properties of borosilicate glass in Table 1 were imported into the Anycasting simulation. Heat transfer coefficient between casting and die was set to 2093.5 W/m^2^·K. Since the heat transfer coefficient between glass and casting was hard to measure, it was set to be the same as that between the mold and casting. Standard continuum surface force (CSF) model was enabled, and RNG (Renormalization Group) k-ε turbulence model was adopted. Nowadays, RNG k-e turbulence model is an optional module in the Anycasting software and is widely used to deal with turbulence flow in die casting. The RNG k-ε turbulence model can be applied to the near wall region directly, and the turbulent vortex is taken into account in this model. Therefore, the RNG k-e turbulence model is suited to simulate the jetting fluid and the flow around circular cylinders under the die casting condition.

## 3. Results and Discussion

### 3.1. The Differences in the Flow Patterns between the Actual Filling Process and the Numerical Simulation

**Figure 3:**
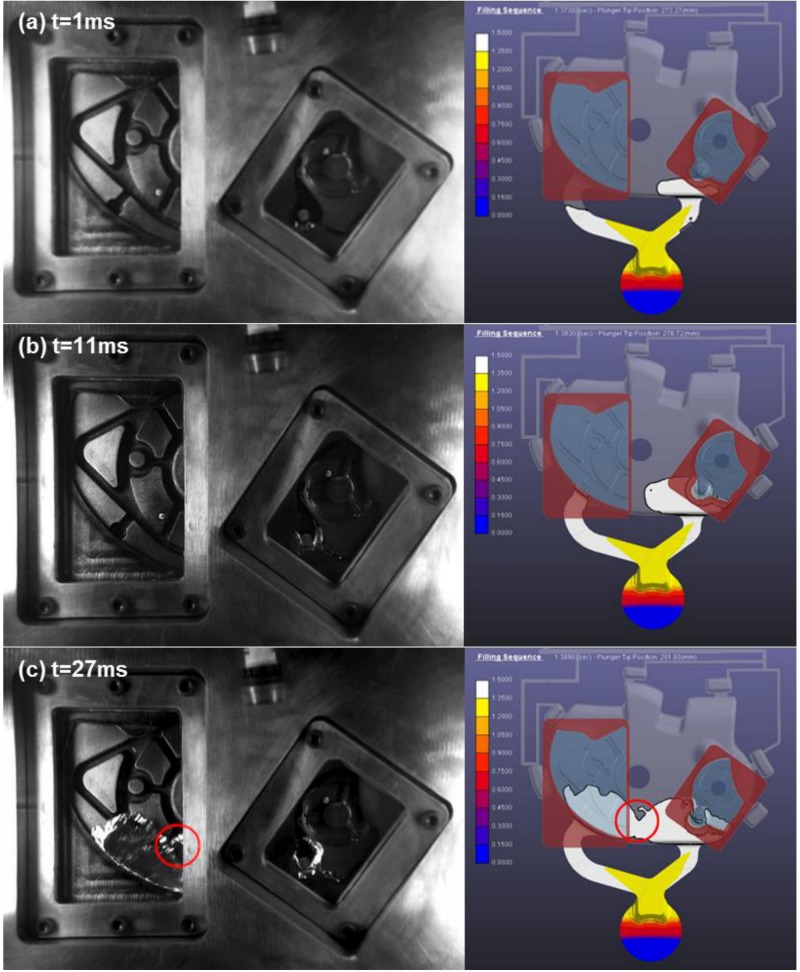

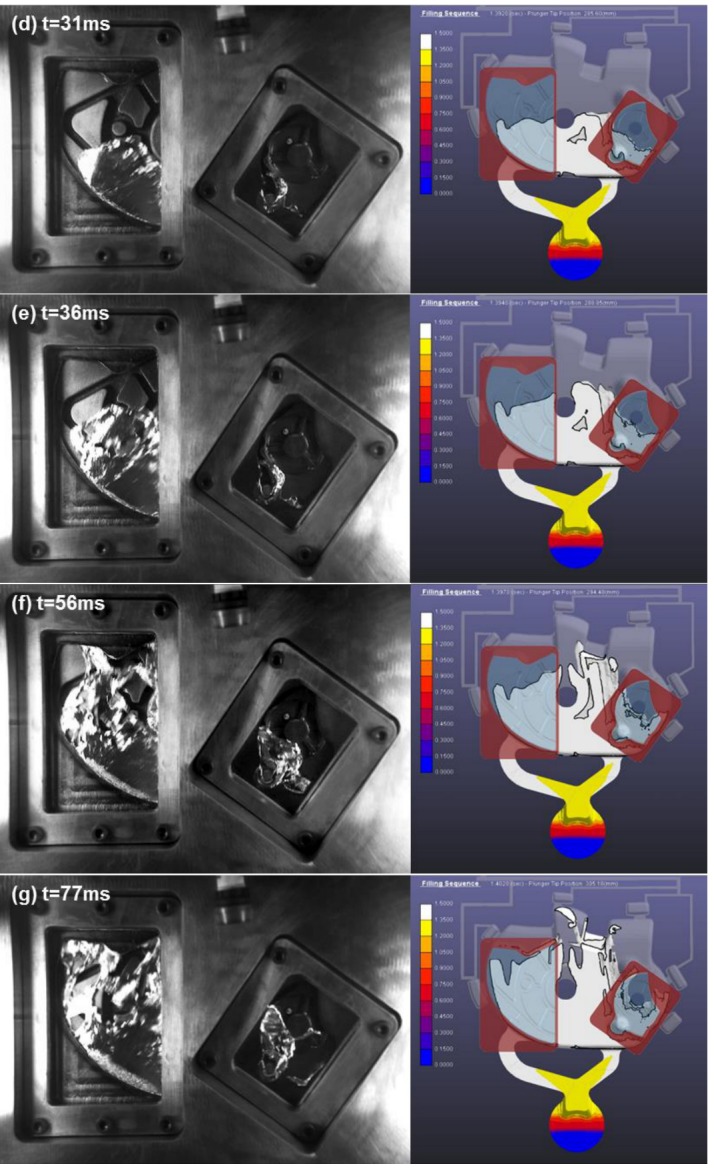

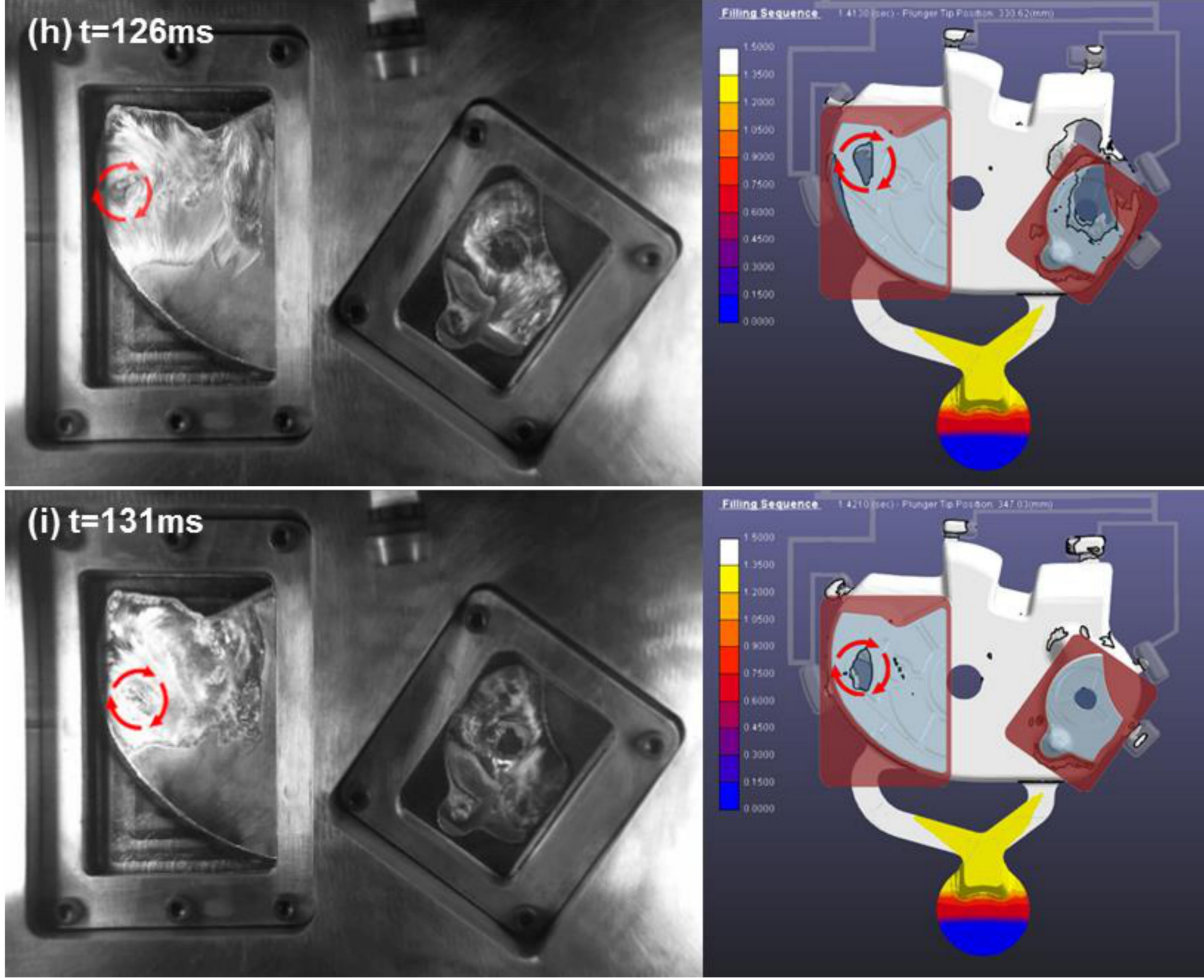
The comparison between the numerical simulation (right) results and the flow visualization experiment (left) within the transparent windows (the time shown in (**a**–**i**) is the actual experimental time from when the fluid has just started entering the right window).

In this paper, the filling process observed under the injection velocity of 2.34 m/s is taken as an example to study the difference in flow pattern between the actual filling process and the numerical simulation results. Figure 3 shows the comparison between numerical simulation (right) results and the flow visualization experiment (left) within the transparent windows. The first frame is determined when the fluid has just started entering the right window in two cases. Then, the difference of flow pattern between the two in the same position is observed.

In the first frame (at 1 ms), the fluid has just started entering the right windows in two cases. The volume of fluid that has entered the window is slightly small for Anycasting. By 11 ms (Figure 3b), this thin (in the third dimension) jet strikes the surface of the raised cylindrical section and starts to flow both up and around its sides in the right window. Due to the low speed at the moment, the flow behavior presents a typical detour flow. This flow pattern shows almost no variance between the two flows. At 27 ms (Figure 3c), two streams of the melt from two ingates both encounter. It can be speculated that the region that two streams of fluid encounter is almost identical in two cases. However, a wave from the right ingate enters the left window (marked by the red circle) in the actual experiment while this region is totally outside the left window in the numerical simulation. It is extremely easy to produce some defects such as cold shut and air pockets in this region. By 31 ms (Figure 3d), the liquid metal fills smoothly and is continuous in both cases. Nevertheless, the flow front is irregular in the actual experiment while the flow front is smooth in the Anycasting simulation.

**Figure 4:**
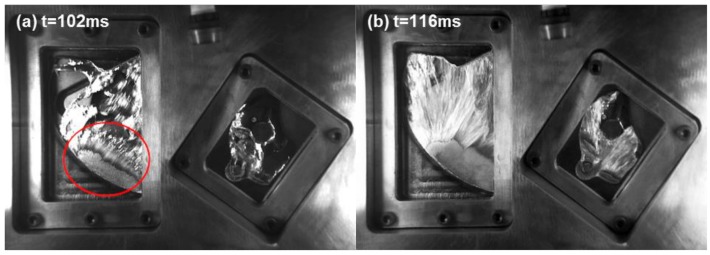
The actual filling process within the transparent windows (the time shown in (**a**,**b**) is the same as Figure 3).

In Figure 3e–g, the significant differences start to appear in the flow pattern between the actual experiment and the Anycasting solution. In the actual experiment, many fragments emerge in the left window. Disintegration leads to an increase of the surface area for the liquid aluminum, which inevitably results in an increase of oxidation. Oxide film can impact the surface tension of melt, heat transfer and so on. In addition, these fragments cool rapidly, and their surfaces partially or completely solidify. When two disintegrated parts collide, it is extremely easy to produce some defects such as cold shut. Moreover, it is liable to trap small amounts of air around their asperities when they pack together. However, almost no fragment is observed in the Anycasting solution. At 104 ms (Figure 4a), the injection process is in the fast shot phase. A jet of high speed (indicated by the red circle) begins to appear in the left window. At 118 ms (Figure 4b), high gate velocities create an atomization phenomenon. When a liquid jet is subjected to the high shear forces between the jet and surrounding gas, the jet disintegrates into some fragments or atomizes. The shear forces mainly depend on the relative velocity between the jet and the gas at the gate discharge [16]. When the injection velocity is high enough, the atomization phenomenon would be observed. However, the atomization phenomenon is not observed in the Anycasting solution.

In Figure 3h, it shows that the last area to be filled is in the left side of the left window. When the fluid jet reaches the top wall of die, it immediately fills back the lower empty region and joins the incoming jet. Thus, a recirculation zones is created, and a clockwise vortex is developed there as marked by the red circle in Figure 3h. This intense vortex would create a near vacuum condition inside it, and any dissolved or free gases around this region will be sucked into this vortex, which induces void and leaves a porous region. By particle tracking in the Anycasting simulation, a clockwise recirculation is also observed at the same position in the Anycasting solution, but the volume of empty region is slightly larger and its interface is clear and smooth. At 131 ms (Figure 3i), the center of vortex moves down in the actual experiment, and the dynamic evolution of air entrainment is clearly observed. Although the vortex also moves downward, its size is different in the Anycasting simulation.

In short, the Anycasting solution is able to make quite accurate predictions for the motion of fluid fronts in the very early stages. As the injection process is in the second injection phase in the very early stages, the velocity of aluminum melts is low and the flow is laminar flow. Moreover, the last area to be filled could also be precisely forecasted. Nevertheless, the significant differences start to appear in the flow pattern between the actual experiment and the Anycasting solution after the break-up and disintegration process takes place. The fragment or atomization formations are the main reasons for the difficulties of modelling the filling process using numerical simulation. 

### 3.2. Porosity Prediction by Direct Observation of Filling Process in the Left Window

The simulation accuracy still needs to improve because of complicated phenomena of the filling process, such as fragment and atomization. The flow visualization experiment can directly observe the actual and real-time filling process by transparent windows installed on the die in this paper, which could provide the reference to predict the porosity at different positions in HPDC.

**Figure 5:**
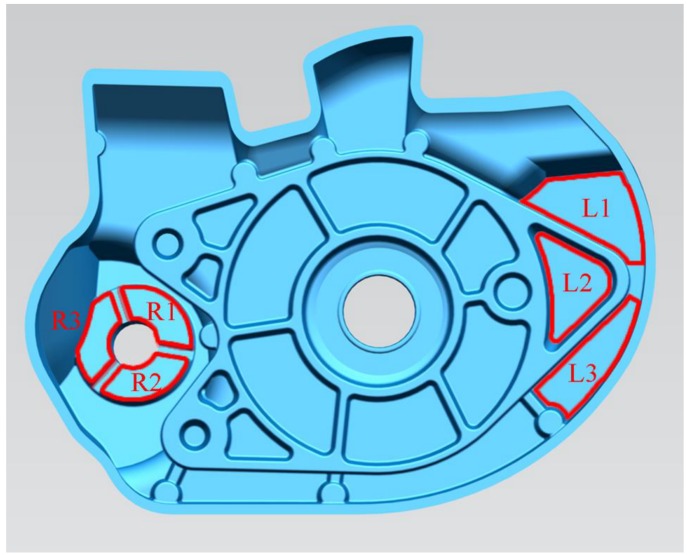
The sampling location (L1, L2, and L3 is located in the left window, and R1, R2, and R3 is located in the right window).

To validate the prediction results, the sampling locations of die-castings under different fast shot speeds are shown in Figure 5. The porosity was measured by hydrostatic weighing method. All specimens are weighted in air and in water respectively, and then their densities are determined according to the following formula:

(1) $$\rho_{p}=\frac{m_{1}}{m_{1}{-m}_{2}}\cdot \rho_{w}$$

where: ρ_p_ and ρ_w_ is, respectively, the density of the specimen and water (unit: g/cm^3^); m_1_ and m_2_ is the mass of the specimen in air and in water respectively (unit: g). Next, the porosity of the examined specimens is calculated from the following relationship:

(2) $$P=\left(1-\frac{\rho_{p}}{\rho_{\mathrm{wz}}}\right)\cdot 100\%$$

where: ρ_wz_ is true density (unit: g/cm^3^), equal to 2.7 g/cm^3^.

**Figure 6:**
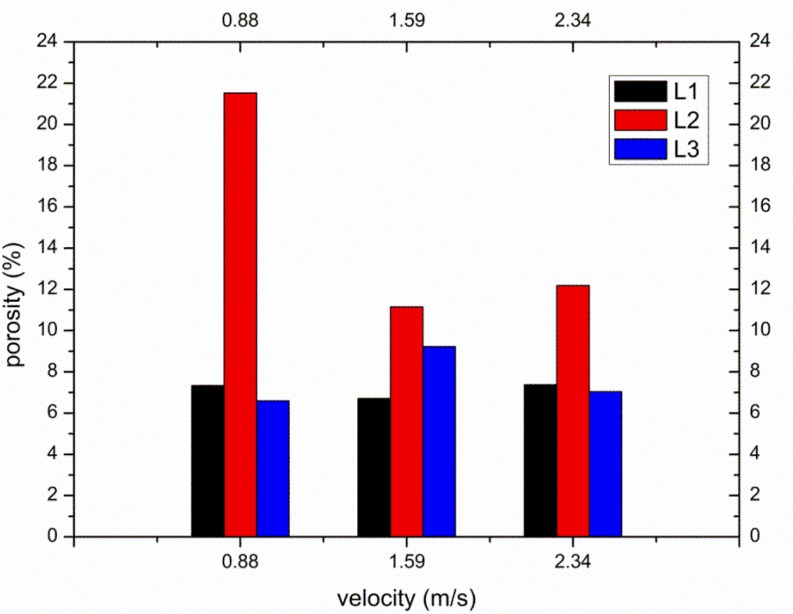
The porosity at different locations in the left window under different fast shot velocities.

**Figure 7:**
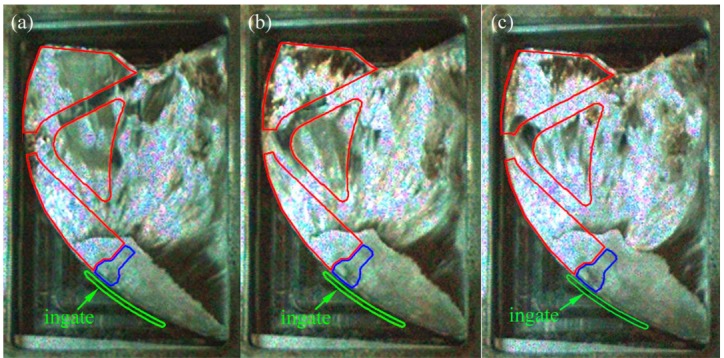
Flow pattern under the fast shot velocity of 0.88 m/s in the left window. (**a**–**c**) are arranged according to the order of filling.

Figure 6 shows the porosity of die-castings at different locations in the left window under different fast shot speeds. From Figure 6, the porosity of the L2 sample is obviously higher than that of other samples, especially under 0.88 m/s. Furthermore, there are small differences between the porosity of the L1 and L3 samples. Figure 7 shows the flow pattern under the fast shot velocity of 0.88 m/s in the left window. Liquid aluminum passes through the ingate at high speed, and it is hindered by the cylindrical hole (the area surrounded by green lines in Figure 7), which results in the flux and velocity of liquid aluminum being reduced above the cylindrical hole. Consequently, there is a vacant land that is not occupied by aluminum melt at L1 and L2 regions, as shown in Figure 7a.

Liquid metal flows forward from both sides of the cylindrical hole with less resistance, and a back flow towards the L2 region is observed when liquid metal collides with the top wall of die and builds up, as shown in Figure 7b. Sequentially, the back flow from the top wall meets the incoming flow from the ingate at the symmetrical lines of the L2 region, and the front of incoming flow is jagged. The returning fluid collided with the filling stream, which would create the turbulence and increase gas entrapment [5]. The fast shot velocity of 0.88 m/s is relatively low, so the temperature and velocity of aluminum melt is very low where two streams of liquid join. Moreover, the collisions between two streams cause turbulence, and turbulence can promote the formation of droplets, which tend to freeze rapidly [17]. Therefore, the back flow and incoming flow are not closely combined, and the L2 sample is filled with a fairly sparse liquid metal (shown in Figure 7c), which creates a large number of pores. Furthermore, liquid metal collides with the top wall of die and heaps up at the L1 region, which would also cause the potential risk of gas entrapment to increase at the L1 region. In addition, air entrainment is not found in the L3 sample. Hence, the porosity of the L1 sample (7.333%) is higher than that of the L3 sample (6.593%) and is lower than that of the L2 sample (21.519%).

**Figure 8:**
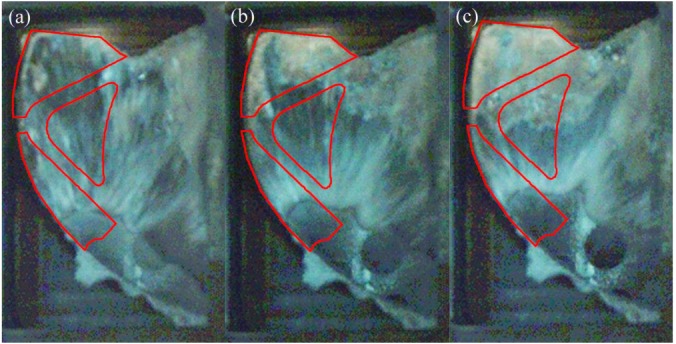
Flow pattern under the fast shot velocity of 1.59 m/s in the left window. (**a**–**c**) are arranged according to the order of filling.

Figure 8 shows the flow pattern under the fast shot velocity of 1.59 m/s in the left window. It is observed that this flow pattern is similar to that under the fast shot velocity of 0.88 m/s. The liquid metal is also diverted by the cylindrical hole when it passes through the ingate, which generates an unoccupied space by aluminum melt at L1 and L2 regions, as shown in Figure 8a. Liquid melt on both sides of the L2 region collides with the top wall of die and meets in the L1 region, which would involve a large number of pores, as shown in Figure 8b. However, compared with the fast shot velocity of 0.88 m/s, there is a larger unoccupied space by aluminum melt before the back flow joining with the incoming flow. This area includes both the L2 region and part of the L3 region, as opposed to including only the L2 region under the fast shot speed of 0.88 m/s, as shown in Figure 8b. It is the main reason that the higher the gate speed, the more focused the aluminum liquid is that reaches the left window plane. Therefore, under the fast shot velocity of 1.59 m/s, only a very narrow stream of liquid aluminum could flow upward from the left side of the cylindrical hole, which results in that part of L3 region not being filled. Moreover, the position where the incoming flow meets the back flow is lower than the symmetrical lines of the L2 region, as shown in Figure 8c, so the area of the junction is reduced in the L2 sample relative to under the fast shot velocity of 0.88 m/s. This is because the back flow has more momentum under the fast shot velocity of 1.59 m/s. Besides, due to more momentum and less heat dissipation, two streams of liquid bond more closely when they join. Therefore, the porosity of L2 sample is lower than that under the fast shot velocity of 0.88m/s. In addition, part of the L3 region is located at the junction of two streams of fluid. Hence, under the fast shot velocity of 1.59 m/s, the porosity of the L3 sample (9.222%) is higher than that of the L1 sample (6.704%) and is lower than that of the L2 sample (11.148%).

**Figure 9:**
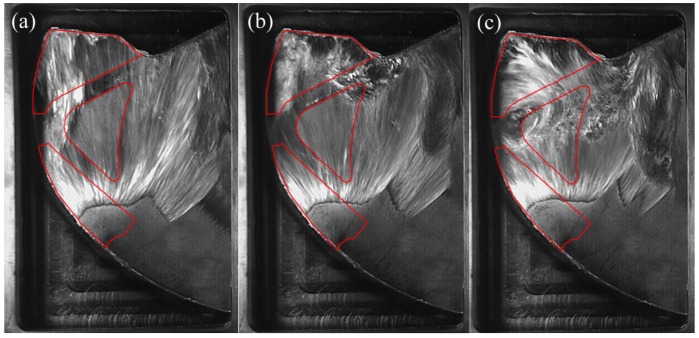
Flow pattern under the fast shot velocity of 2.34 m/s in the left window. (**a**–**c**) are arranged according to the order of filling.

Figure 9 shows the flow pattern under the fast shot velocity of 2.34 m/s in the left window. It exhibits that this flow pattern is nearly identical to that under the fast shot velocity of 1.59 m/s. There is an unoccupied space by aluminum melt at L1 and L2 regions, as shown in Figure 9a. Liquid metal collides with the top wall of die and accumulates at the L1 region. Both the L2 region and a small fraction of the L3 region is not filled with liquid aluminum before the back flow joining with the incoming flow, as shown in Figure 9b. Nevertheless, compared with the fast shot velocity of 1.59 m/s, the position where the incoming flow meets the back flow is higher. This is because although the velocity of back flow increases, the rate at which the cylindrical hole is filled also increases under the fast shot velocity of 2.34 m/s. Consequently, the blocking effect of the cylinder hole fails in advance, and the back flow and the incoming flow meet at the symmetrical lines of the L2 region. The area of junction becomes bigger in the L2 sample, which results in that the porosity of L2 sample is higher than that under the fast shot velocity of 1.59 m/s. In addition, the L3 region is not located at the junction of two streams of fluid, as shown in Figure 9c. Therefore, the porosity of the L1 sample (7.370%) is higher than that of the L3 sample (7.037%) and is lower than that of the L2 sample (12.185%).

### 3.3. Porosity Prediction by Direct Observation of Filling Process in the Right Window

**Figure 10:**
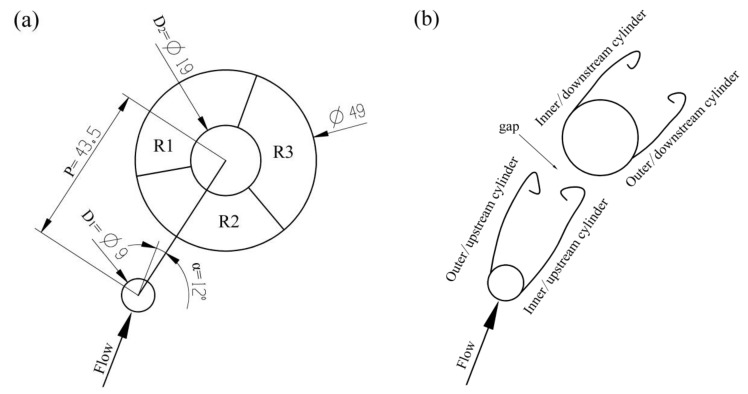
Schematic diagram showing (**a**) the arrangement of the two cylinders in the right window; (**b**) shear layer designations.

**Figure 11:**
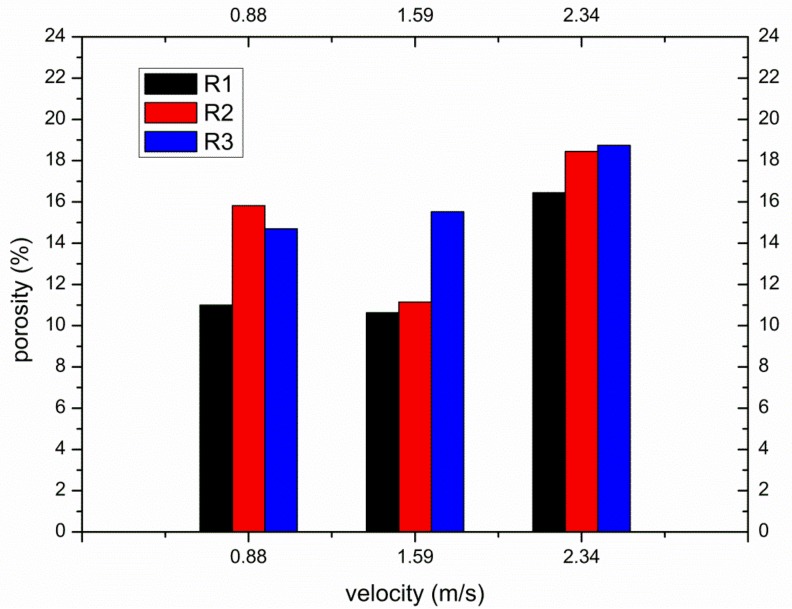
The porosity at different locations in the right window under different fast shot velocities.

**Figure 12:**
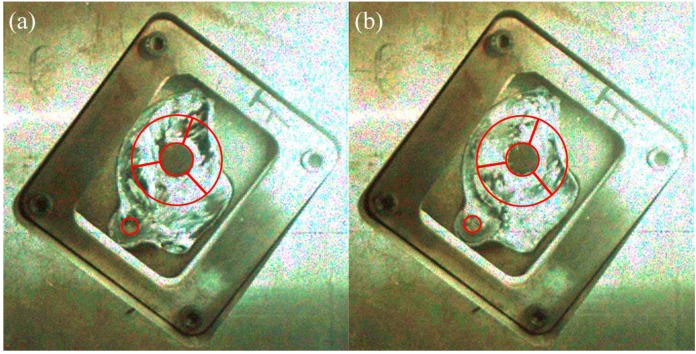
Flow pattern under the fast shot velocity of 0.88 m/s in the right window. (**a**,**b**) are arranged according to the order of filling.

According to the flow pattern observed in the flow visualization video, the flow in the right window could be simplified down to the planar flow around two staggered cylinders. Figure 10 shows the arrangement of the two cylinders in the right window. Figure 11 shows the porosity of die-castings at different locations in the right window under different fast shot speeds. When the fast shot speed is 0.88 m/s, the relationship of porosity could be descript as the R2 sample is highest; the R3 sample is next; while R1 sample is lowest. From Figure 12, it is observed that the inner separated shear layer of upstream cylinder reattaches to the outer surface of the downstream cylinder, whereas the outer separated shear layer of the upstream cylinder does not contact the downstream cylinder. In addition, although there is a gap between the two cylinders, the gap is surrounded by the reattached shear layer on one side and by the outer shear layer of the upstream cylinder on the other side, which effectively prevents the incoming flow from penetrating this gap. Consequently, almost no oncoming flow runs through the gap, and there is only nearly stagnant fluid in the gap. 

Sumner et al. [18] summarized the nine low subcritical regime flow patterns for two staggered circular cylinders of equal diameter in cross-flow. Under the fast shot velocity of 0.88 m/s, the flow pattern in the right window is similar to the “shear layer reattachment flow pattern (SLR)” above. Besides, Gu and Sun [19] made also similar discoveries for two staggered circular cylinders in the high subcritical Reynolds numbers, and denominated the flow pattern as “Pattern I_B_.” The principal part of R3 sample is submerged in the wake of the large cylinder, as shown in Figure 12a. A two-eddy configuration pattern is observed in the near-wake region, and a rapid increase in the frequency of vortex shedding when the flow is under the Reynolds numbers [20]. A large negative pressure appears in the wake, and the maximum turbulent intensity is located on the near-wake region [21], which would result in that large quantities of gases are sucked and involved. The main part of the R2 sample is located in the near-wake region of upstream cylinder, and R2 sample is filled later than R3 sample, as shown in Figure 12b, which causes the porosity of R2 sample to be higher than that of the R3 sample. The incoming flow, which is close to the outer separated shear layer of upstream cylinder, reaches the inner surface of the downstream cylinder directly (R1 region), so the R1 region is almost free from the interference of wake. Therefore, under the fast shot velocity of 0.88 m/s, the porosity of the R3 sample (14.704%) is higher than that of the R1 sample (11.000%) and is lower than that of the R2 sample (15.815%).

**Figure 13:**
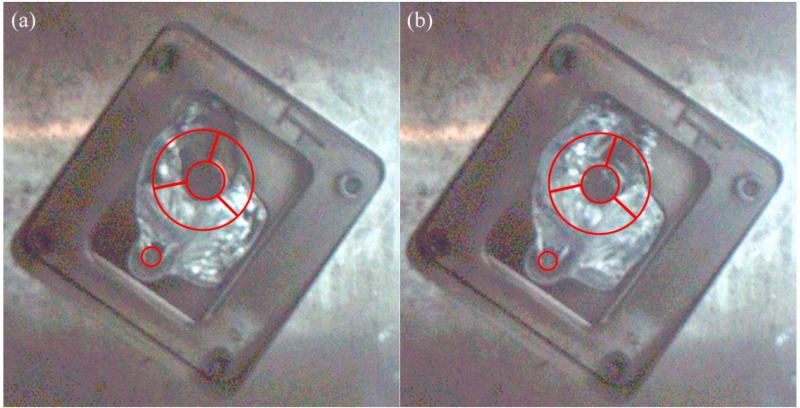
Flow pattern under the fast shot velocity of 1.59 m/s in the right window. (**a**,**b**) are arranged according to the order of filling.

Under the fast shot speed of 1.59 m/s, the relationship of porosity is: the R3 sample is highest; next is the R2 sample; the R1 sample is lowest between them in the right window. From Figure 13, the near-wake region becomes narrow behind the upstream cylinder and deviates from the downstream cylinder and the flow axis. Meanwhile, the inner separated shear layer of the upstream cylinder has no capacity to reattach to the outer surface of the downstream cylinder, and the oncoming flow is allowed to penetrate the gap between the cylinders. This flow pattern is similar to “induced separation flow pattern (IS)” denoted by Sumner et al. [18]. This pattern is also similar to “Pattern II_B_” denominated by Gu and Sun [19]. The near-wake region is highly compressed by the gap flow, and the R2 sample keeps away from this region, so the wake of the small cylinder has only a little effect on the porosity of the R2 sample. Moreover, the R3 region situated in the rear of downstream cylinder is filled last. The oncoming flow closed to the outer separated shear layer of upstream cylinder reaches the R1 region directly and runs through the gap between the cylinders up to the R1 region. Hence, the porosity of the R2 sample (11.148%) is higher than that of the R1 sample (10.630%) and is lower than that of the R3 sample (15.519%).

The Reynolds number plays an important role in the flow pattern around a circular cylinder. It is well known that the boundary layer in the front of the circular cylinder undergoes a transition from laminar to turbulent when Reynolds numbers approach 2 × 10^5^ [22]. In the critical regime (2 × 10^5^ < Re < 3.5 × 10^5^) [23], the turbulence transition in the boundary layer makes the pressure difference between the rear of the cylinder and front less, and the drag force reduce suddenly. When the fluid goes around this cylinder, it has more inertia and is able to go around a little bit more, which causes the delay of the separation point of the shear layer and a little bit more compact wake. In addition, in the supercritical regime (3.5 × 10^5^ < Re < 1.5 × 10^6^) [23], the boundary layer becomes turbulent completely, and the drag force around the cylinder begins to recover. The Reynolds number (Re = UD_1_/ν) is defined in terms of the incoming flow velocity U, the upstream cylinder diameter D_1_ and the kinematic viscosity of fluid ν. Under the three fast shot velocities, Reynolds numbers are 1.9 × 10^5^ (0.88 m/s), 3.3 × 10^5^ (1.59 m/s), and 4.9 × 10^5^ (2.34 m/s), respectively. Therefore, in the same arrangement of the two cylinders, when the fast shot velocity increases from 0.88 to 1.59 m/s, the turbulence transition takes place in the boundary layer and the shear layer is delayed to separate from the upstream cylinder. Thereby, the relationship of porosity of three samples also changes from R1 < R3 < R2 (0.88 m/s) to R1 < R2 < R3 (1.59 m/s).

**Figure 14:**
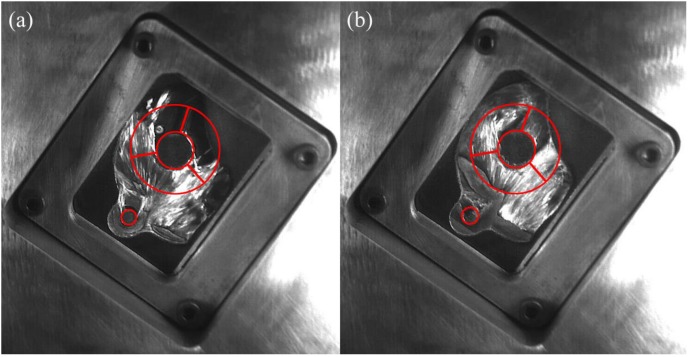
Flow pattern under the fast shot velocity of 2.34 m/s in the right window. (**a**,**b**) are arranged according to the order of filling.

When the fast shot speed increases to 2.34 m/s, the flow pattern at the moment is the same as that under the fast shot velocity of 1.59 m/s, as shown in Figure 14. The near-wake region is highly confined in the gap between the two cylinders, and the incoming flow is allowed to pass through the gap. Consequently, the relationship of porosity is identical to that under the fast shot velocity of 1.59 m/s and could be descript as the R3 sample is highest; next is the R2 sample; the R1 sample is lowest between them. 

## 4. Conclusions

In this paper, the actual filling process of HPDC is directly observed by the flow visualization experiment. The differences in flow pattern between the actual filling process and the Anycasting software solution are compared. Meanwhile, the porosity of samples at different locations is predicted under three different fast shot velocities. We draw the following conclusions from our investigations:

(a) The flow visualization experiment can clearly observe the whole die casting filling process, including complex flow phenomena, such as fragment and atomization. Therefore, it could be a valuable tool for validating experimentally the outcome of flow simulations.

(b) The significant differences start to appear in the flow pattern between the actual experiment and the Anycasting solution after the fragment or atomization formation. In addition, the dynamic evolution of air entrainment is clearly observed, the volume of the empty region is small, and its interface is rough in the flow visualization experiment while the volume of the empty region is slightly larger and its interface is clear and smooth in the Anycasting solution.

(c) According to direct observation of the filling process, the porosity distribution of die-castings can be accurately predicted. The fast shot velocity would determine the position at which the back flow from the top of die meets the incoming flow from the inner gate. The junction of two streams of fluid would create more porosity than other locations. There is a transition in flow patterns due to drag crisis under high fast shot velocity around two staggered cylinders, which result in the porosity relationship also changing from R1 < R3 < R2 (0.88 m/s) to R1 < R2 < R3 (1.59 and 2.34 m/s). Meanwhile, the region submerged in the wake of circular cylinder would produce greater porosity than other regions.

## Figures and Tables

**Table 1:** Properties of borosilicate glass.

Properties	Value	
Density	2.23 g/cm^3^	
Bending Strength	120–160 MPa	
Thermal Expansion Coefficient (20-350 °C)	32-35 × 10^-6^ cm/cm·°C	
Thermal Conductivity (20 °C)	0.82 W/m·°C	
Specific Heat	820 J/kg·°C	
Chemical Composition (wt.%)	SiO_2_	81.0%
	B_2_O_3_	12.5%
	Al_2_O_3_	2.32%
	Na_2_O + K_2_O	6.0%

**Table 2:** Boundary conditions and mesh properties.

Properties	Value
Material	P20 Steel
Special Heat	(285.5 + 1.076 T) J/kg·K
Density	(7920 – 0.4739 T) kg/m^3^
Thermal Conductivity	(46.08 + 0.0022 T) W/m·K
Total Mesh Cells	28,545,644
Mesh Cell Size	0.001702 m

**Table 3:** Thermophysical properties of melt in the simulation.

Property	Value	
Material	Pure Aluminum	
Initial Temperature	750 °C	
Liquidus	675 °C	
Special Heat	900 J/kg·K	
Density	2705 kg/m^3^	
Thermal Conductivity	234 W/m·K	
Latent Heat	389,000 J/kg	
Surface Tension	(−0.0028 T + 2.762) N/m	
Dynamic Viscosity	Temperature	Value
	660 °C	0.003017 kg/m·s
	680 °C	0.002762 kg/m·s
	700 °C	0.002541 kg/m·s
	720 °C	0.002349 kg/m·s
